# Mn (III) tetrakis (4-benzoic acid) porphyrin scavenges reactive species, reduces oxidative stress, and improves functional recovery after experimental spinal cord injury in rats: comparison with methylprednisolone

**DOI:** 10.1186/1471-2202-14-23

**Published:** 2013-03-01

**Authors:** Danxia Liu, Yichu Shan, Lokanatha Valluru, Feng Bao

**Affiliations:** 1 Department of Neurology, University of Texas Medical Branch, 301 University Blvd., Rt. 0881, Galveston, TX 77555-0881, USA; 2 Departments of Biochemistry & Molecular Biology, University of Texas Medical Branch, 301 University Blvd., Rt. 0881, Galveston, TX 77555-0881, USA

**Keywords:** Antioxidant therapy, Behavioral test, Mn (III) tetrakis (4-benzoic acid) porphyrin, Oxidative stress, Reactive oxygen species, Secondary spinal cord injury

## Abstract

**Background:**

Substantial experimental evidence supports that reactive species mediate secondary damage after traumatic spinal cord injury (SCI) by inducing oxidative stress. Removal of reactive species may reduce secondary damage following SCI. This study explored the effectiveness of a catalytic antioxidant - Mn (III) tetrakis (4-benzoic acid) porphyrin (MnTBAP) - in removing reactive oxygen species (ROS), reducing oxidative stress, and improving functional recovery *in vivo* in a rat impact SCI model. The efficiency of MnTBAP was also compared with that of methylprednisolone – the only drug used clinically in treating acute SCI.

**Results:**

*In vivo* measurements of time courses of ROS production by microdialysis and microcannula sampling in MnTBAP, methylprednisolone, and saline (as vehicle control)-treated SCI rats showed that both agents significantly reduced the production of hydrogen peroxide, but only MnTBAP significantly reduced superoxide elevation after SCI. *In vitro* experiments further demonstrated that MnTBAP scavenged both of the preceding ROS, whereas methylprednisolone had no effect on either. By counting the immuno-positive neurons in the spinal cord sections immunohistochemically stained with anti-nitrotyrosine and anti-4-hydroxy-nonenal antibodies as the markers of protein nitration and membrane lipid peroxidation, we demonstrated that MnTBAP significantly reduced the numbers of 4-hydroxy-nonenal-positive and nitrotyrosine-positive neurons in the sections at 1.55 to 2.55 mm and 1.1 to 3.1 mm, respectively, rostral to the injury epicenter compared to the vehicle-treated animals. By behavioral tests (open field and inclined plane tests), we demonstrated that at 4 hours post-SCI treatment with MnTBAP and the standard methylprednisolone regimen both significantly increased test scores compared to those produced by vehicle treatment. However, the outcomes for MnTBAP-treated rats were significantly better than those for methylprednisolone-treated animals.

**Conclusions:**

This study demonstrated for the first time *in vivo* and *in vitro* that MnTBAP significantly reduced the levels of SCI-elevated ROS and that MnTBAP is superior to methylprednisolone in removing ROS. Removal of ROS by MnTBAP significantly reduced protein nitration and membrane lipid peroxidation in neurons. MnTBAP more effectively reduced neurological deficits than did methylprednisolone after SCI - the first most important criterion for assessing SCI treatments. These results support the therapeutic potential of MnTBAP in treating SCI.

## Background

Traumatic spinal cord injury (SCI) is worsened by secondary damage processes caused by the overproduction of endogenous deleterious substances [1,2]. Reactive species (RS), including reactive oxygen species (ROS) and reactive nitrogen species (RNS), are believed to contribute to secondary damage after SCI by oxidatively damaging proteins, DNA, and phospholipids [3-5]. ROS include superoxide anion (O_2_^•-^), hydrogen peroxide (H_2_O_2_) and hydroxyl radical (^•^OH). O_2_^•-^ is produced through several aerobic pathways during normal metabolism, and superoxide dismutase (SOD) converts O_2_^•-^ into H_2_O_2_. H_2_O_2_ is reduced to H_2_O by catalase, glutathione peroxidase (GPx), and thioredoxin. A dynamic equilibrium exists between the potential for oxidative damage and the antioxidant defense capacity. Disruption of this balance produces excessive O_2_^•-^ and H_2_O_2_, which can produce ^•^OH by the metal-catalyzed Haber-Weiss/Fenton reaction [6,7]. ROS attack polyunsaturated fatty acids in cell membranes, triggering free radical chain reactions to cause membrane lipid peroxidation (MLP) to produce aldehydes such as malondialdehyde (MDA) and 4-hydroxy-nonenal (HNE) - the by-products of MLP. HNE damages proteins by forming covalent aldehyde-protein complexes [8]. This compromises protein function by altering secondary and tertiary structure, further impairing cells [9,10]. Excessive O_2_^•-^ can also react with RNS nitric oxide to form powerful oxidant peroxynitrite (ONOO^-^). Peroxynitrite in turn reacts with SOD to form a nitronium-like intermediate, which nitrate tyrosine in proteins to produce nitrotyrosine (Ntyr), a marker of protein nitration [11,12]. Hence, HNE-positive or Ntyr-positive neurons might be functionally impaired and more susceptible to subsequent death.

Substantial experimental evidence supports RS as important mediators of secondary damage after SCI [13-15]. We and others have demonstrated that the levels of O_2_^•-^[16], H_2_O_2_[17], ^•^OH [18], catalytic iron [19], nitric oxide [20,21], ONOO^-^[20,22], and the products of oxidation and nitration of proteins [20,23-26] and MLP [26-29] all significantly increased following SCI. The increased levels of RS are sufficient to cause oxidative damage to major cellular components [30,31], neuronal death by necrosis and apoptosis [32-34], and neurological dysfunction [32] in uninjured rat spinal cords. These results suggest that RS-caused oxidative stress is a major pathway of secondary damage after SCI. Therefore, removing RS with a broad-spectrum RS scavenger may reduce secondary SCI.

Metalloporphyrins, as a novel class of catalytic antioxidants, not only scavenge a wide range of RS such as O_2_^•-^ , H_2_O_2_, ONOO^-^, and lipid peroxyl radicals [35], but also modulate RS-based redox signaling pathways [36]. It was reported that the *in vivo* actions of Mn (III) complexes probably do not depend upon catalysis of the dismutation reaction, but the complex can be reduced by both enzymatic and spontaneous routes, and the resultant Mn (II) complex can be reoxidized by O_2_^•-^ with a rate constant of ~ 4 × 10^9^ M^-1^ S^-1^[37]. The metalloporphyrin Mn (III) tetrakis (4-benzoic acid) porphyrin (MnTBAP) possesses SOD and catalase-like activity [38], and also scavenges ONOO^-^[39]. It is also a potent inhibitor of MLP [40]. MnTBAP not only converts O_2_^•-^ to H_2_O_2_, but it also catalyzes dissociation of H_2_O_2_ to water - the catalase activity; this blocks the Haber-Weiss pathway for ^•^OH production, thereby blocking ROS damage. In the central nervous system, cerebroventricular injection of MnTBAP inhibited kainate-induced mitochondrial O_2_^•-^ production, DNA oxidation and neuronal loss in the hippocampus [41]. We demonstrated *in vivo* that MnTBAP reduced ONOO^-^-induced oxidation and nitration of proteins [30] and MLP [31] in the rat spinal cord. It prevented ONOO^-^- and ^•^OH-induced necrotic and apoptotic cell death [33,34]. These results suggest that the catalytic antioxidant MnTBAP may be beneficial as antioxidant therapy after SCI owing to its cell permeability, low toxicity, and broad scavenging of RS, and therefore warrant closer examination. However, the ROS-reduction ability of MnTBAP has never been tested *in vivo* in an experimental SCI model.

A high-dose regimen of methylprednisolone (MP) improves neurological recovery from SCI in humans [42,43]. MP is the only drug currently used clinically in treating acute SCI and its high-dose regimen has become the standard of treatment. To evaluate the treatment potential of a new candidate, it is critical to compare its efficacy with that of existing treatment agents. The goals of the present study were 1) To evaluate the ability of recently established intrathecal optimal dose of MnTBAP [44] to reduce O_2_^•-^ and H_2_O_2_ produced in the extracellular space of the rat spinal cord following SCI and to compare the ROS-reducing abilities between the optimal dose of MnTBAP and the standard regimen of MP in *in vivo* and *in vitro* experiments. 2) To examine the capability of the optimal dose of MnTBAP to protect against RS-induced neuronal oxidative damage following SCI as an addition to our previous funding that a dose lower than the optimal dose of MnTBAP significantly reduced oxidative stress and neuron loss, but MP had no effect on either [45]. 3) To compare the efficiency between MnTBAP and MP in improving functional recovery by behavioral tests. The resulting data would explore the potential of MnTBAP for antioxidant therapy following SCI.

## Methods

Male Sprague–Dawley rats (200–250 g) were used in all *in vivo* experiments. The procedures in the rats were approved by the University of Texas Medical Branch Animal Care and Use Committee and are in accord with the National Institutes of Health’s *guide for the Care and Use of Laboratory Animals*. Every effort was made to minimize animal suffering and the number of animals used.

### Examination of the ability of MnTBAP and MP to remove ROS after SCI

To evaluate the ability of MnTBAP and MP to reduce H_2_O_2_ and O_2_^• ─^ produced after SCI, the time courses of O_2_^•-^ and H_2_O_2_ productions in the extracellular space of the rat spinal cord were measured by microdialysis and microcannula sampling respectively, according to our published methods [16,17] in three groups of rats treated with MnTBAP, MP, and saline (vehicle control) upon injury (n = 6 for each group). The procedures for anesthesia, animal surgery, preparation and insertion of microdialysis fiber and microcannula, microdialysis and microcannula sampling, and impact injury are described in detail in our previous publications [16,17,20].

#### Animal preparation

The rats were anesthetized with sodium pentobarbital (35 mg/kg body weight) intraperitoneally (ip), followed by urethane (500 mg/kg) to maintain anesthesia until sacrifice. After the rats were fully anesthetized, a laminectomy was performed on vertebrae T12–L1. A pin was inserted into one end of a microdialysis fiber or a microcannula and glued in place by using SuperGlue (Kwik·Fix, Chemence,Inc.; Alpharetta, GA, USA). The pin was pushed laterally through the gray matter of the cord at T13, and the fiber or cannula was pulled through the cord following the pin until the trailing marker just disappeared into the cord. This placed the dialysis zone or the holes in the gray matter of the cord. The dorsal half of the L5 vertebra was also removed, and a small hole was made in the exposed dura. A perfusing catheter (200 μm inside diameter and 300 μm outside diameter, 11 kDa molecular weight cutoff; Filtral AN 69-HF, Hospal Industrie, Meyzieu, France) was inserted through the hole 1 cm caudal to the terminal cistern in the rat intrathecal space as described previously [46]. There was enough space to implant the catheter without injuring the nerves and there was no cord in this area that might be damaged by the catheter. The animal was then clamped in a frame by attachment to its dorsal vertebral processes. Body temperature was maintained at 37–38°C throughout the experiment by utilizing feedback from a rectal probe to a heating blanket (Harvard homeothermic blanket control unit; Les Ulis, France). The pin was then cut off and the microdialysis fiber or microcannula was attached to a microdialysis pump (CMA, Microdialysis AB, Solna, Sweden) with a length of polyethylene (PE)-50 tubing for sampling. The catheter was also attached to another syringe pump through a PE-20 tube for drug delivery.

The microdialysis fibers or microcannulae were made as follows: Two 1 mm wide ink marks flanking a 2-mm area were made on a microdialysis fiber (Spectra/Por RC; 200 μm inside diameter, Spectrum Laboratories, Inc, Houston, TX, USA). Then, the fibers were coated with a thin layer of silicone rubber (3140 MIL-A-46146 RTV, Dow Corning Corporation, Midland, MI, USA) with a final external diameter of 220 μm. For sampling H_2_O_2_, the 2-mm dialysis zone was uncoated [17]. For measuring O_2_^•-^ production, two pairs of holes were made within the 2-mm zone through the wall of a completely coated fiber, allowing administered cytochrome c to diffuse through the holes owing to the concentration gradient, as we previously described [16].

#### Microdialysis and microcannula sampling and impact injury

A pool was formed around the exposed spinal cord. Mineral oil (37°C) was poured into the pool, and the temperature of the oil was maintained at 36–37°C by utilizing feedback from a thermosensor placed in the oil pool to a heating lamp set above the pool (Physitemp Instruments G15, Inc., Clifton, NJ, USA). Artificial cerebrospinal fluid (ACSF), composition in mM: Na^+^ 151.1, K^+^ 2.6, Mg^2+^ 0.9, Ca^2+^ I.3, C1^-^ 122.7, HCO_3_^-^ 21.0, HPO_4_^2-^ 2.5 and glucose 3.87.) was pumped through the fiber at a rate of 5.0 μl/min or the cannula at a rate of 15.0 μl/min. ACSF was bubbled with 95% O_2_/5% CO_2_ prior to each experiment to adjust the pH to between 7.0 and 7.4. Sample collection was begun 75 min after insertion of the fiber or cannula into the cord, allowing the release of substances owing to the insertion of the fiber or cannula to subside to a stable baseline. Fluid was collected from the outlet end of the fiber or cannula in plastic vials in ice. After 60 min of fluid collection at 20 min per sample to obtain a basal level, the mineral oil was removed and the spinal cord was injured over the zone containing the fiber or cannula by dropping a 10-g rod through a guide tube 2.5 cm down to the cord (25 g·cm injury force), originally developed by Allen [47]. After impact injury of the cord, the pool was refilled with mineral oil and samples were collected over 20 min intervals for 5 h.

The New York University (NYU) spinal cord impactor, designed based on the weight drop method, is a standard device for an impact injury [48]. However, in the above experimental procedures, the microdialysis fiber or microcannula, and the drug delivery catheter have to be implanted into the rat and connected to pumps, and the rat has to be connected to a heating blanket and a heating lamp, an oil pool has to be made around the exposed cord, and basal samples have to be collected prior to injury. Therefore it was impossible to use the NYU device to perform an injury for these types of experiments. The validation of our weight drop method was demonstrated in our previous publications using microdialysis or microcannula sampling [16,17,20].

#### Drug administration

It was reported that MnTBAP poorly penetrates the blood–brain barrier [49]. Therefore, we established an optimal intrathecal dose of 4 mg/kg MnTBAP for its ability to protect against MLP and neuron death [44]. In the present study, the established optimal dose was administered through the implanted catheter into the intrathecal space of the rat spinal cord in the MnTBAP-treated group. MnTBAP was dissolved in 0.1 M NaOH, the solution was diluted with 0.9% saline, and the pH was adjusted to 7.1-7.3 [49]. Due to the low solubility of MnTBAP, 10 mg/mL MnTBAP was administered approximately one hour before injury so that the optimal dose of MnTBAP could be completely administered at the time point of impact injury at a rate of 1.5 μl/min.

Methylprednisolone sodium succinate (MPSS), a water-soluble derivative of MP, is the form used in animal experiments and in clinical treatments of SCI. MPSS quickly breaks down to MP, the effective component of MPSS, following its administration. The standard regimen of MPSS, given intravenously (iv), consists of 30 mg/kg as a bolus dose followed by 5.4 mg/kg/h as a maintenance dose. The maximum MP concentration ratio of cerebrospinal fluid to blood is nearly 20% following iv administration [50]. According to this ratio, a 6 mg/kg bolus dose and a 1.08 mg/kg/h maintenance dose of MPSS given intrathecally equals the standard clinical regimen of MPSS given iv. Therefore, in the MPSS-treated group, a 6 mg/kg bolus dose was administered into the intrathecal space of the rat spinal cord over a 10-min period and was completed at the time point of impact injury. Then the 1.08 mg/kg/h maintenance dose was administered continually for three hours starting at 2 hour post-SCI. A total of 9.24 mg/kg MPSS was administered into the intrathecal space of every rat at a rate of 1.5 μl/min.

### Examination of the ability of MnTBAP and MP to remove ROS *in vitro*

To compare the abilities of MnTBAP and MP to scavenge H_2_O_2_*in vitro*, three groups of experiments were conducted: 2.5 μM H_2_O_2_ treated with ACSF, with 20 mg/L MnTBAP, and with 30 mg/L MPSS, as shown in Table 1. All reagents were dissolved in ACSF. The final volume of solution was 400 μl. In each group, three samples of identical composition were prepared. After incubation of the mixture of H_2_O_2_ and drugs at 37°C for 30 min, 100 μl of each sample was mixed with 100 μl of water containing 1 mM salicylate and 0.2 mM FeCl_2_ and allowed to react for 5 min. The Fe^2+^ catalyzed dissociation of the remaining H_2_O_2_ to produce ^•^OH by the Fenton reaction: Fe^2+^ + H_2_O_2_ → Fe^3+^ + ^•^OH + OH^-^. The produced ^•^OH attacks salicylate to produce 2,3- and 2,5- dihydroxybenzoic acid (2,3- and 2,5-DHBA), which were measured by high-pressure liquid chromatography (HPLC), as described below. Comparison of 2,3-DHBA concentrations between control and MnTBAP- or MPSS-treated samples established the ability of these drugs to scavenge H_2_O_2_*in vitro*.

**Table 1:** ***In vitro *removal of H**_**2**_**O**_**2 **_**by MnTBAP and MPSS**

**Sample**	**Reagent added**				**Total volume**	**Final concentrations**
	**H**_**2**_**O**_**2**_	**MnTBAP**	**MPSS**	**ACSF**		
	**(5.0 μM)**	**(40 mg/L)**	**(60 mg/L)**			
Control	200 μl	0	0	200 μl	400 μl	2.5 μM H_2_O_2_
MnTBAP-treated	200 μl	200 μl	0	0	400 μl	2.5 μM H_2_O_2_
						20 mg/L MnTBAP
MPSS-treated	200 μl	0	200 μl	0	400 μl	2.5 μM H_2_O_2_
						30 mg/L MPSS

The abilities of MnTBAP and MPSS to scavenge O_2_^•-^ were also compared *in vitro*. As for measuring H_2_O_2,_ three groups of experiments were similarly performed as shown in Table 2. All reagents were dissolved in ACSF and the final volume of solution for each sample was 400 μl. In each group, three samples of identical composition were prepared. O_2_^•-^ was generated by the xanthine and xanthine oxidase pair as reported [51] and as we previously demonstrated *in vivo*[18]. O_2_^•-^ generation was started by adding xanthine to a solution containing xanthine oxidase and cytochrome c in ACSF in the vial. Xanthine oxidase catalyzed xanthine to produce O_2_^•-^, and the O_2_^•-^ generated in turn reduced cytochrome c to reduced cytochrome c. The absorbance of reduced cytochrome c was measured spectrophotometrically at 2 min following addition of xanthine, as described below. Comparison of the absorbance of reduced cytochrome c between control and MnTBAP- or MPSS-treated samples provided the ability of these drugs to scavenge O_2_^•─^*in vitro*.

**Table 2:** ***In vitro *removal of O**_**2**_^**•- **^**by MnTBAP and MPSS**

**Sample**	**Reagent added**						**Total volume**	**Final concentrations**
	**Cytochrome c (200 μM)**	**Xanthine oxidase**	**Xanthine (80 μM)**	**MnTBAP (80 mg/L)**	**MPSS (120 mg/L)**	**ACSF**		
		**(100 unit/L)**						
Control	100 μl	100 μl	100 μl	0	0	100 μl	400 μl	50 μM cytochrome c, 25 unit/L xanthine oxidase, 20 μM xanthine
MnTBAP-treated	100 μl	100 μl	100 μl	100 μl	0	0	400 μl	50 μM cytochrome c, 25 unit/L xanthine oxidase, 20 μM xanthine, 20 mg/L MnTBAP
MPSS-treated	100 μl	100 μl	100 μl	0	100 μl	0	400 μl	50 μM cytochrome c, 25 unit/L xanthine oxidase, 20 μM xanthine, 30 mg/L MPSS

### Analysis of H_2_O_2_ and O_2_^•-^ in microdialysates, perfusates, and *in vitro* reactants

H_2_O_2_ in the collected microdialysates were measured by converting the sampled H_2_O_2_ to ^●^OH in the collecting vials by the Fenton reaction using a unique method developed in our laboratory [17]. A solution containing FeCl_2_ (0.2 mM) and salicylate (1 mM) in water (pH 3.0) were pre-added to the collecting vial (1:1, microdialysate : solution). H_2_O_2_ sampled through the microdialysis fiber immediately reacts with FeCl_2_, while reaching the solution in the collecting vials to generate ^●^OH in the vials by the Fenton reaction. The ^●^OH produced in the collecting vials rapidly attacks salicylate to produce 23- and 2,5-DHBA. The DHBAs in the collecting vials in the *in vivo* and in the reactants in the *in vitro* experiments were measured by HPLC with electrochemical detection [52]. A Shimadzu (Shimadzu Scientific Instruments Inc., Columbia, MD, USA) HPLC (LC-10AD pump, SIL-10AXL auto injector) with a Phenomenex C18 (3-mm particle, 15 cm × 4.6 mm, Phenomenex U.S.A., Torrance, CA, USA) analytical column and an ESA Coulochem II electrochemical detector (ESA, Inc., Chelmsford, MA, USA) were used in this experiment. The voltages of the guard cell and analytical electrode were 750 mV and 250 mV, respectively. The mobile phase was 0.03 M citric acid, 0.03 M sodium acetate, and 20% methanol at an elution flow rate of 0.7 ml/min. 2,3-DHBA as a specific marker of ^● ^OH production was calibrated to the concentration of H_2_O_2_ by using a standard curve of [H_2_O_2_] vs [2,3-DHBA], as previously described in detail [17].

The levels of O_2_^•-^ in the extracellular space of the cord were measured by perfusing cytochrome c through a microcannula inserted into the gray matter of the cord and measuring reduced cytochrome c by using the method that we developed previously [16]. After the initial ACSF perfusion, the perfusing fluid was changed to a cytochrome c solution (50 μM in ACSF) to capture the O_2_^•-^ in the extracellular space of the cord. The collected perfusates were centrifuged at 12,000 × g for 10 min to precipitate blood and tissue fragments. The absorbance of reduced cytochrome c in the supernatant in the *in vivo* experiments and in the reactants in the *in vitro* experiments was measured by a BioSpec-1601 spectrophotometer (Shimadzu Scientific Instruments, Inc., Columbia, MD, USA) at a characteristic absorbance wavelength of 550 nm. The perfusing fluid (cytochrome c solution) in the *in vivo* experiments or the mixture without adding xanthine in the *in vitro* experiments was used as the reference in the measurement [16].

### Examination of the ability of MnTBAP to protect against oxidative stress after SCI

In the experiments for examining the effect of MnTBAP protection against oxidative stress, two groups of rats treated with MnTBAP or saline as vehicle control (n = 4 for each group) were used.

#### Animal preparation, drug administration and tissue processing

The rats were anesthetized with sodium pentobarbital (50 mg/kg, ip) without maintenance doses. Following a laminectomy on vertebra T13, the cords were injured by using the standard NYU spinal cord impactor. A 10 g rod was dropped 1.25 cm onto the exposed cord (12.5 g.cm force) using the NYU device. Immediately after SCI, approximately 20 μl (depending on the weight of the rat) of MnTBAP (4 mg/kg in saline) or saline as vehicle control was administered through the implanted catheter into the intrathecal space and completed within approximately 10 min. After injury and drug administration, the incision was surgically repaired. The body temperature of the rat was maintained by using a heating pad during surgery and until the rat awakened.

At 24 h post-SCI, the rat was re-anesthetized and perfused transcardially with saline followed by 4% paraformaldehyde in phosphate-buffered saline (PBS, pH 7.2-7.4). Following the perfusion, 15-mm segments centered at the injury site were removed and fixed in the same fixative for another 24 h at 4°C. The fixed cords were dehydrated and embedded in paraffin. The serial sections (10 μm thickness) were cut with a microtome and mounted on glass slides coated with poly-L-lysine (five sections per slide) for immuno-staining. Every 10th slide in the series was deparaffinized and stained with 0.1% cresyl violet (ACROS, New Jersey, USA) in sodium acetate buffer for 30 min [34]. These sections were observed under a light microscope to locate the section with the lowest neuron density as the injury epicenter (defined as distance zero).

#### Immunohistochemical staining

To evaluate oxidative damage, HNE and Ntyr, as markers of MLP and protein nitration respectively, were used for immunohistochemical staining according to our published procedures [30,31,45]. The primary antibodies were HNE polyclonal antiserum (1:100, rabbit-anti-HNE, from Alpha Diagnostic Intl., San Antonio, TX, USA) and anti-Ntyr (1:500, mouse-anti-Ntyr, from Cayman Chemical, Ann Arbor, MI, USA). The secondary antibody was biotinylated goat-rabbit-IgG or biotinylated goat-mouse-IgG respectively (Invitrogen, Camarillo, CA, USA). Ready-to-use histostain-plus streptavidin-horse radish peroxidase (Invitrogen, Camarillo, CA, USA) was used to label biotinylated antibodies, then sections were stained with a liquid 3,3’-diaminobenzidine substrate kit (Invitrogen, Camarillo, CA, USA).

HNE- and Ntyr-positive neurons were also visualized by immuno-fluorescence-double staining with antibodies against HNE and neuron-specific enolase (NSE) or Ntyr and NSE. In brief, the sections were immuno-stained with a first primary antibody, rabbit-anti-HNE (1:100, Alpha Diagnostic Intl., San Antonio, TX, USA), or rabbit anti-Ntyr (1:200, Cayman chemical, Ann Arbor, MI, USA) and labeled fluorescently with a first secondary antibody, anti-rabbit Alexa Fluor 488 (green, Invitrogen, Camarillo, CA, USA). After the sections were incubated in goat serum for 30 min, the sections were immuno-stained with a second primary antibody, mouse-anti-NSE (1:100, Dako Cytomation, Glostrup, Denmark) and then labeled with a second secondary antibody, anti-mouse Alexa Flour 594 (red, Invitrogen, Camarillo, CA, USA). These sections were washed in PBS three times and cover slipped with Pro-Long Gold anti-fade reagent (Invitrogen, Camarillo, CA, USA). A fluorescence microscope (Olympus model BX-51) was used for photomicrographs with green (AF-488) and red (AF-594) filters.

#### Cell counting

MnTBAP protection against oxidative stress was evaluated spatially by counting the immuno-stained neurons on the sections at different distances rostral to the epicenter. The images of immunohistochemically stained sections were observed under a light/fluorescence microscope (Olympus BX51) and captured by a digital camera (Olympus DP70 microscope digital camera) with a computer system and software for processing photographs. HNE- and Ntyr-positive neurons were identified based on the positively immuno-stained cytoplasm with bright nuclei and the morphology of neurons. The positively immuno-stained neurons in the gray matter of the cord in each section were counted in areas of the ventral left and ventral right quarters. Quarters were defined by horizontal and vertical lines drawn crossing the central canal in the center of the sections. This area of gray matter includes motoneurons, middle- and small-sized neurons, and glial cells. The neurons in each left or right quarter were counted three times and the counts averaged for that quarter; then the counts in the left and right quarters were averaged for that section. To avoid bias, the person processing the cord into sections and the person counting the cells in the sections were blinded. This counting method has been validated by our previous publications [20,44,45,53] and by those of many others.

### Evaluation of the ability of MnTBAP and MP protection against neurological dysfunction after SCI

SCI causes hind limb paralysis and inability of the rat to support its body weight, so the scrotum of male rats touched and dragged around the bottom of cage - increasing the incidence of infection. Therefore, in most publications using behavioral tests to study SCI, female rats are used. This study examined the efficacies of MnTBAP and MPSS in improving neurological deficits in four groups of female rats (200–225 g): sham control (N=4), SCI treated with MnTBAP, MPSS or saline as vehicle control (N=8 for the three injured groups). SCI-induced neurological deficits were evaluated by the Basso-Beattie-Bresnahan locomotor rating scale (BBB-test) and the inclined plane test for 10 weeks. In the sham-operated control group, animals underwent the same surgical procedures but no impact injury was performed. All experiments were triple blinded.

#### Animal preparation, drug administration and impact injury

The rats were given 0.1 mg/kg buprenorphine subcutaneously (s.c.) 15 min prior to anesthesia and then anesthetized with pentobarbital (50 mg/kg, i.p.). After a laminectomy, the exposed cords were injured on vertebra T10 by a 10 g rod dropping 2.5 cm onto the exposed cord (25 g.cm force) using the NYU device. Then, the muscle and skin were sutured and 10 ml saline was injected (ip) to prevent dehydration. The body temperature of the rat was maintained using a heating pad during surgery and until waking up. To avoid an additional incision on L5 for intrathecal drug delivery which would affect the accuracy of behavioral tests, drugs were administered by iv or ip injection for behavioral tests. We previously demonstrated that MnTBAP (10 mg/kg) given by ip injection significantly reduces neuron loss [53]. In the present study, 10 mg/kg of MnTBAP or saline was given ip for behavioral tests. The standard regimen of MPSS established for clinical treatment of SCI was given by iv injection. The first dose of MnTBAP (10 mg/kg) or MPSS (30 mg/kg) was given at 4 h post-SCI followed by one half of the first dose 2 h later. These rats were housed in a temperature and humidity controlled room with a 12 h light/dark cycle. In the first week following SCI, the bladder was emptied manually until the voiding reflex was recovered; buprenorphrine (0.5 mg/kg, s.c.) was given post-injury to minimize the impairment or discomfort of injured rats. The body weight of the animal was monitored for 10 weeks during behavioral tests. Since only the hindlimbs were paralyzed, the rat could still reach food and water.

#### BBB-test

The motor function of rats was assessed using the BBB locomotor rating scale as reported [54,55]. The test is designed to assess progressive locomotor recovery after SCI. The open field is simply a circular pool of 1 meter in diameter. In order to observe animal locomotion, pretest training of animals was required. Before injury, the rats were exposed to the testing environment twice a day for 5 days. Each rat was handled several times during each 30–60 min session so that they no longer showed signs of fear, and became accustomed to being picked up and walked continuously in the open field environment. Then the rats were individually placed in the open field for 4 min to assure that all subjects consistently obtained a maximum score of 21. After SCI, the animals were evaluated for hind limb motor function, either alone for 4 min or in pairs for 5 min, at day 3, and weekly from 1 – 10 weeks by two blinded observers. Briefly, this scale involves closely monitoring limb movement, weight-bearing capability, coordination, and gait with scores ranging from 0 (complete paralysis) to 21 (normal locomotion) corresponding to fully coordinated and proper gait, movement at all joints, weight support capability, and appropriate limb, body, and tail positioning. Two examiners were positioned across from each other to observe both sides of the rat.

#### Inclined plane test

The rat was placed on an inclined plane covered by a rubber mat (with 0.6 mm deep grooves) constructed as reported [56,57]. The plane can be adjusted to different slopes to measure the animal's ability to maintain its body position for 5 sec without falling. The animal was placed on the board three times at each of two positions: right side up and left side up. The average score for the two positions was used. The angle of the board was increased from 0° until the rat could only maintain its position for 5 seconds. Final readings to the nearest 5° were taken for the stationary positions. All animals were assessed at day 3, and every week from 1 to 10 weeks after SCI.

### Statistical analysis

Data were expressed as mean ± SEM. Two-way repeated measures analysis of variance (RMANOVA) was used to compare the time courses of ROS elevation between MnTBAP- and saline-treated, MPSS- and saline-treated, and between MnTBAP- and MPSS-treated groups. Two-way RMANOVA followed by all pairwise multiple comparison (Bonferroni t-test) was also used to compare the differences between MnTBAP and saline treatment in protection against MLP and protein nitration, and the differences between MnTBAP and saline, MPSS and saline, MnTBAP and MPSS in reducing neurological dysfunction. One-way analysis of variance (ANOVA) followed by the Tukey test was used to compare the differences in the levels of ROS among different treatment groups in *in vitro* experiments. The paired t-test was used to assess differences between average post-injury and average pre-injury H_2_O_2_ and O_2_^•-^ levels in their time courses of production. P < 0.05 was considered a statistically significant difference.

## Results

### MnTBAP versus MP on reducing elevated ROS following SCI

#### MnTBAP and MP both reduced SCI-elevated H_2_O_2_ concentrations

**Figure 1:**
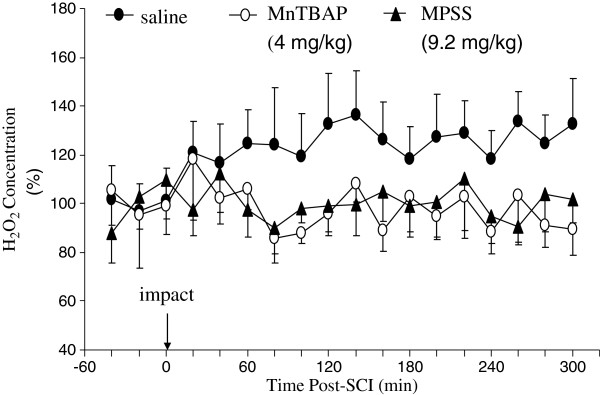
**The time courses of extracellular H**_**2**_**O**_**2 **_**concentration changes following impact injury to the rat spinal cord in saline-treated, MPSS-treated, and MnTBAP-treated groups. **The procedure was described in detail in the Methods section. After three microdialysates were collected at 20-min intervals to establish a basal level, a weight was dropped at time 0 onto the cord (25 g·cm), and sampling was continued for another 5 h at 20-min intervals. A single dose of MnTBAP (4 mg/kg) or the equivalent intrathecal standard regimen of MPSS (bolus dose plus maintenance dose for a total of 9.24 mg/kg as described in the Methods) was administered into the intrathecal space of the cord through a catheter implanted in the terminal cistern. The samples were collected in vials containing a solution of FeCl_2 _and salicylate. H_2_O_2 _in the microdialysates were converted to ^● ^OH in the collecting vials by the Fenton reaction. The ^● ^OH produced in the collecting vials rapidly attacks salicylate to produce 2,3- and 2,5-DHBA. The salicylate hydroxylation products 2,3- and 2,5-DHBA were analyzed by HPLC with electrochemical detection. The 2,3-DHBA was calibrated to the amount of H_2_O_2_. Clearly, both MnTBAP and MP reduced H_2_O_2_ to its basal level.

The time course of H_2_O_2_ elevation following SCI was measured *in vivo* by microdialysis sampling and HPLC analysis. The abilities of MnTBAP and MP to reduce SCI-elevated H_2_O_2_ were evaluated by administering the optimal dose of MnTBAP (4 mg/kg) and the equivalent standard regimen of MPSS ((bolus dose plus maintenance dose for a total of 9.24 mg/kg as described in the Methods) into the intrathecal space of the rat spinal cord. Figure 1 illustrates the time courses of H_2_O_2_ concentration changes in the extracellular space following SCI in saline-treated, MnTBAP-treated, and MPSS-treated groups. Owing to the variation of H_2_O_2_ concentrations among animals, the H_2_O_2_ concentrations measured in the microdialysates were normalized: the average basal level of H_2_O_2_ from the three pre-injury time points for each animal was considered to be 100, and the post-injury concentration measured at each time point was presented as the percentage of the basal level. The H_2_O_2_ levels in each rat during the basal period (time points 1–3) and during the post-SCI period (time points 4–18) were averaged and a paired t-test was performed to compare the differences in H_2_O_2_ levels between post-injury and pre-injury in their time courses of production. In the saline-treated group, the levels of H_2_O_2_ increased in the first post-injury sample (20 min) and remained elevated for 5 h. The average H_2_O_2_ level during the post-trauma period (116 ± 2.5%, mean ± SEM) was statistically significantly higher (p=0.005) than the average pre-injury H_2_O_2_ level (100.0 ± 5.1%). In the MnTBAP-treated group, the average level of H_2_O_2_ in the post-trauma period was 100.3 ± 2.5% (mean ± SEM) of the average pre-injury level (100.0 ± 6.7%, mean ± SEM), not significantly different (p=0.9). In the MPSS-treated group, the average level of H_2_O_2_ in the post-trauma period was 100.4% ± 2.7% (mean ± SEM) of the average pre-SCI level of H_2_O_2_ (100.0 ± 6.3%, mean ± SEM), also not significantly different (p=0.9). This indicates that treatment by the optimal dose of MnTBAP and the standard regimen of MPSS both significantly reduced the post-SCI H_2_O_2_ concentration to its pre-SCI levels – a 100% reduction.

Two-way RMANOVA was used to compare the differences of post-injury levels of H_2_O_2_ between different treatment groups. The post-injury H_2_O_2_ levels in both MnTBAP-treated and MPSS-treated groups were significantly lower than those for the saline-treated group (p<0.001 for both agents). There were no significant differences in post-injury levels of H_2_O_2_ between MPSS-treated and MnTBAP-treated groups (p=1.0). MnTBAP (4 mg/kg) and MPSS (9.24 mg/kg) administered intrathecally both significantly reduced the level of H_2_O_2_ in the extracellular space to the basal level, indicating a similar ability to attenuate elevated H_2_O_2_ following SCI.

#### MnTBAP, but not MP reduced SCI-elevated O_2_^•-^ concentrations

**Figure 2:**
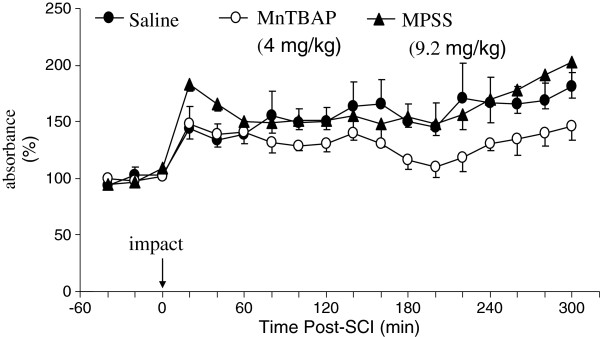
**The time courses of extracellular O**_**2**_^• **─ **^**concentration changes following impact injury to the rat spinal cord in saline-treated, MPSS-treated, and MnTBAP-treated groups. **The procedure was described in detail in the Methods. O_2_^• ─^ was captured by perfusing cytochrome c through the microcannula. After three samples were collected at 20-min intervals to establish the basal level, a weight was dropped at time 0 onto the cord (25 g·cm) and sampling was continued for another 5 h at 20-min intervals. MnTBAP and MPSS were administered similarly, as in Figure 1. The perfusates were collected, centrifuged, and the absorbance of reduced cytochrome c was measured spectrophotometrically. MnTBAP, but not MP, significantly reduced O_2_^• ─^ levels.

The time course of O_2_^•-^ elevation following SCI was measured *in vivo* by microcannula sampling the O_2_^•-^ -reduced cytochrome c and measuring the absorbance of reduced cytochrome c spectrophotometrically. The abilities of MnTBAP and MP to reduce SCI-elevated O_2_^•-^ were similarly evaluated, as for H_2_O_2_. Figure 2 illustrates the time course of O_2_^•-^ level changes in the extracellular space of the cord following SCI in the same three groups of rats, as for H_2_O_2_. The levels of O_2_^•-^ were similarly normalized and statistically analyzed, as for H_2_O_2_. In the saline-treated group, the levels of O_2_^•-^ increased in the first post-injury sample and remained elevated for 5 h. The average level of O_2_^•-^ in the post-trauma period (157 ± 4.1%, mean ± SEM) was significantly higher (p<0.001) than the average pre-injury O_2_^•-^ levels (100.0 ± 2.9%, mean ± SEM). In the MnTBAP-treated and MPSS-treated groups, the average post-SCI levels of O_2_^•─^ were 132 ± 4.7% (mean ± SEM) and 165 ± 4.3% (mean ± SEM) respectively, which were significantly higher than the average pre-injury levels of O_2_^•-^ (100.0 ± 1.4% and 100.0 ± 2.6%, p<0.001).

Comparison of the time course of post-injury levels of O_2_^•-^ in the extracellular space of the cord between saline-treated and drug-treated by RMANOVA demonstrated that the optimal dose of MnTBAP significantly reduced post-injury O_2_^•-^ levels (p<0.001). However, there was no significant difference in the post-injury O_2_^•-^ levels between saline-treated and MPSS-treated groups (p=0.3). The post-injury O_2_^•-^ levels in the MnTBAP-treated group were also significantly lower than those for the MPSS-treated group (p<0.001). The optimal dose of MnTBAP significantly reduced the elevation of O_2_^•-^ by approximately 50% compared with the vehicle- treated group, whereas the standard regimen of MP did not attenuate the elevation of O_2_^•─^.

### MnTBAP versus MP on scavenging ROS *in vitro*

**Figure 3:**
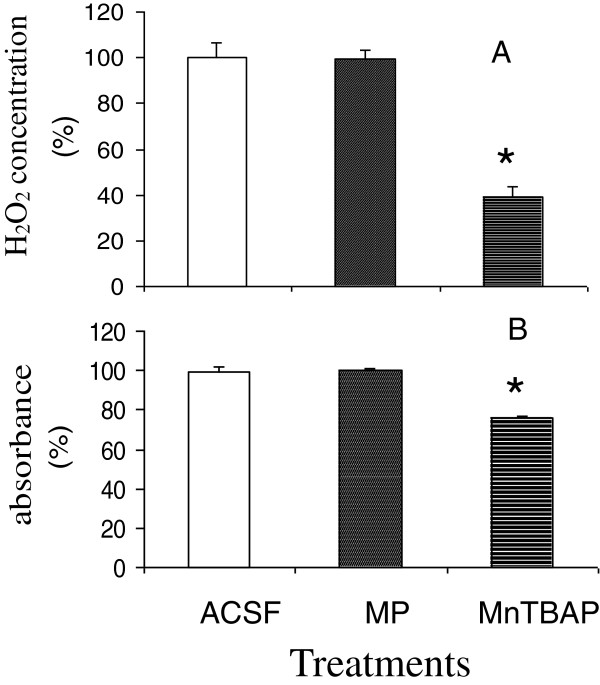
***In vitro *comparison of the abilities of MnTBAP and MP to remove H**_**2**_**O**_**2 **_**(A) and O**_**2**_^• **─ **^**(B). **The experimental groups were described in the Methods section and in Tables 1 and 2. The levels of H_2_O_2 _and O_2_^• ─ ^were measured by HPLC or spectrophotometrically, as described in Figures 1 and 2. MnTBAP, but not MPSS, significantly reduced both H_2_O_2 _and O_2_^• ─^ levels.

*In vitro* testing was completed to explore the mechanism of different ROS removal ability between MnTBAP and MP. Figure 3 represents the concentrations of H_2_O_2_ and O_2_^•-^ measured in *in vitro* experiments. The concentrations of H_2_O_2_ and O_2_^•-^ in the MnTBAP- or MPSS-treated groups were normalized as the percentage of the ACSF-treated group. One-way ANOVA followed by the Tukey test was used to compare the differences in the levels of H_2_O_2_ and O_2_^•-^ among different treatment groups. (A) The concentration of H_2_O_2_ in the MnTBAP-treated group (38.7 ± 3.4%, mean ± SEM) was significantly lower than that in the ACSF-treated group (100.0 ± 4.8%, mean ± SEM, p<0.001) and in the MPSS-treated group (99.1 ± 3.0%, mean ± SEM, p<0.001). There was no significant difference (p=0.9) in the concentrations of H_2_O_2_ between MPSS-treated and ACSF-treated groups. The *in vitro* experiment demonstrated that MnTBAP significantly reduced H_2_O_2_ levels to less than 40% of the levels of vehicle treatment, whereas MP did not reduce H_2_O_2_. (B) The O_2_^•-^ levels in the MnTBAP-treated group (76.8 ± 0.8%, mean ± SEM) were significantly lower than those in the ACSF group (100.0 ± 2.3%, mean ± SEM, p<0.001) and in the MPSS-treated group (100.8 ± 0.8%, mean ± SEM, p<0.001). There was no difference in the O_2_^•-^ levels between MPSS-treated and ACSF-treated groups (p=0.9). This *in vitro* experiment demonstrated that MnTBAP significantly reduced O_2_^•-^ levels to less than 80% of the levels in the vehicle-treated group, but MPSS did not reduce O_2_^•-^ levels. The O_2_^•-^ levels in the MnTBAP-treated group were significantly lower than those in the MPSS-treated group (p<0.001). It can be concluded that MnTBAP directly scavenges both H_2_O_2_ and O_2_^•-^; however, MPSS had no effect on levels of either H_2_O_2_ or O_2_^•-^ compared with their levels in the ACSF-treated group.

### MnTBAP protects against oxidative stress after SCI

We have previously compared the antioxidative abilities of MnTBAP with the standard regimen of MP, and demonstrated that MnTBAP at a dose of 2.5 mg/kg significantly reduced MLP, protein nitration and neuron loss, but MP had no effect on either [45]. The present study evaluated the optimal dose of MnTBAP protection against oxidative stress in neurons. The spatial profiles of oxidative stress in neurons were established by counting the HNE- and Ntyr-positive neurons in the spinal cord sections immunohistochemically stained with anti-HNE and anti-Ntyr antibodies at different distances from the epicenter. Protection by MnTBAP was evaluated by comparing the counts in the sections treated with the optimal dose of MnTBAP and with saline as vehicle controls using two-way RMANOVA followed by all pairwise multiple comparison (Bonferroni t-test).

**Figure 4:**
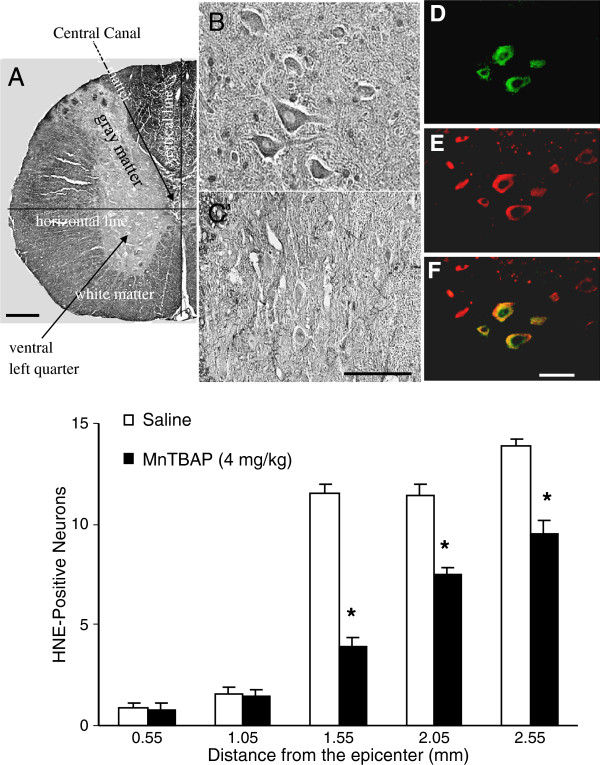
**MnTBAP protects against MLP. **The animal experiment, spinal cord injury, and tissue processing were described in the Methods section. After the epicenter was located, the sections 0.55 to 2.55 mm rostral to the epicenter were immuno-stained at 0.5-mm intervals with an anti-HNE antibody. Upper panel, photomicrographs of anti-HNE antibody immuno-stained sections. **A**, lower magnification of a HNE stained spinal cord section showing the morphology of cord section and indicating the cell counting area defined by horizontal and vertical lines drawn crossing the central canal in the center of the sections, as described in *cell counting *in the Methods section. **B **and **C**, higher magnification of HNE stained spinal cord sections at 1.55 mm rostral to the epicenter. **B**, saline-treated and **C**, MnTBAP-treated sections. The colored panels (**D**-**F**) show double-immuno-fluorescence staining with anti-HNE (**D**, green) and anti-NSE (**E**, red) antibodies, and immuno-colocalization of **D **and **E **(**F**, yellow). The color photomicrographs are at a higher magnification. Scale bars are 100 μm. Lower panel, spatial profile of MnTBAP protection against MLP. The HNE-positive neurons were counted, and the counts compared between MnTBAP-treated and the vehicle-treated animals at different distances from the epicenter. MnTBAP significantly (indicated by an asterisk*) reduced the number of HNE-positive neurons in the sections 1.55 to 2.55 mm from the epicenter. Error bars, mean ± S.E.M.

Figure 4 (upper panel) provided photomicrographs of anti-HNE immuno-stained sections 1.55 mm rostral to the epicenter in MnTBAP- and saline-treated groups. The MnTBAP-treated section (C) had fewer HNE-positive neurons compared with the saline-treated group (B). D-F presented images of a section double-immuno-fluorescence-stained with anti-HNE and anti-NSE antibodies in a MnTBAP-treated cord to verify the presence of HNE-positive neurons. The neurons stained with NSE (E) showed normal morphology. The same sections stained with HNE (D) showed not only a reduced number of HNE-positive neurons compared with E, but also partial HNE staining in some HNE-positive neurons because MnTBAP attenuated MLP in neurons. The double-stained MLP in neurons was co-localized (F). Therefore, anti-HNE immuno-staining did not stain entire neurons. MnTBAP protection against MLP was quantified by counting numbers of HNE-positive neurons in the sections 0.55 to 2.55 mm rostral to the epicenter in MnTBAP- and saline-treated groups. Comparison of the counts between the two treatment groups demonstrated that 4 mg/kg of MnTBAP significantly (P<0.001) decreased the number of HNE-positive neurons in the ventral gray matter of the cord. The number of HNE-positive neurons in the MnTBAP-treated group was significantly decreased (P<0.001 for all) at 1.55 (2.1 ± 0.7), 2.05 (7.5 ± 0.32) and 2.55 (9.6 ± 0.6) mm compared with vehicle-treated sections at 1.55 (7.6 ± 1.2), 2.05 (11.4 ± 0.59), and 2.55 (13.8 ± 0.41) mm rostral to the epicenter (Figure 4 lower panel).

**Figure 5:**
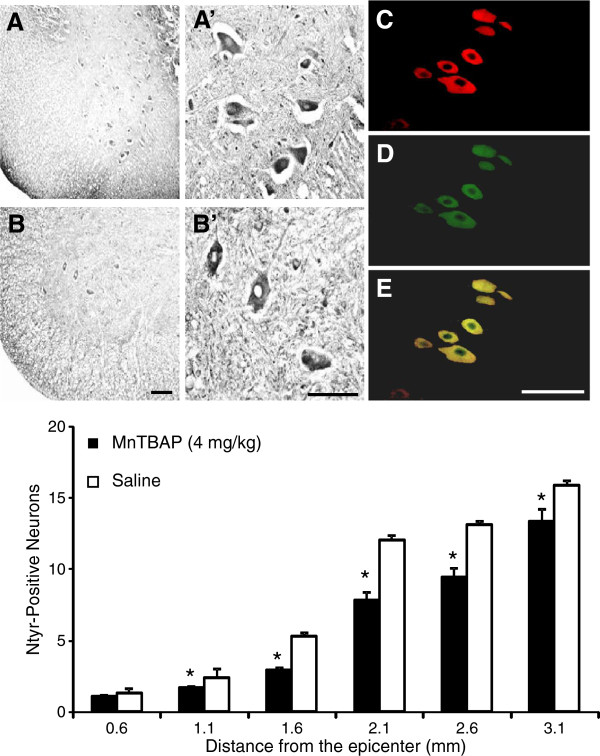
**MnTBAP protects against protein nitration. **The sections 0.6 to 3.1 mm rostral to the epicenter in the animals, as shown in Figure 4, were immuno-stained at 0.5-mm intervals with an anti-Ntyr antibody. Upper panel, photomicrographs of rat spinal cord sections 2.6 mm rostral to the epicenter immuno-stained with an anti-Ntyr antibody. **A**-**B**, lower magnification; A’-B’, higher magnification of **A**-**B**. A-A’, saline-treated and B-B’, MnTBAP-treated sections. Fewer Ntyr-positive neurons appeared in the MnTBAP-treated sections. **C**-**E**, immuno-colocalization of a double-immuno-fluorescence-stained section with an anti-Ntyr antibody (**D**, green) and an anti-NSE (**C**, red) antibody, and immuno-colocalization of **C **and **D** (**E**, yellow). Scale bars100 μm. The lower panel presents the quantitative results of MnTBAP protection against protein nitration obtained by counting Ntyr-positive neurons in the sections at different distances from the epicenter. Comparison of the counts between the sections treated by optimal dose of MnTBAP and those treated by vehicle indicates that MnTBAP significantly (indicated by an asterisk*) reduced the number of Ntyr-positive neurons in the sections 1.1 to 3.1 mm to the epicenter. Error bars, mean ± S.E.M.

Figure 5 (upper panel) presents anti-Ntyr-immuno-stained sections 2.6 mm rostral to the epicenter in saline-treated (A) and MnTBAP-treated (B) groups. The MnTBAP-treated section had fewer Ntyr-positive neurons and more normal morphology of neurons compared with the saline-treated section. The identities of the Ntyr-positive neurons were further verified by double-immuno-fluorescence staining with anti-Ntyr (D ) and anti-NSE (C) antibodies from a saline-treated section 2.6 mm from the epicenter, and the double-stained nitrated proteins in neurons were co-localized (E). MnTBAP protection against protein nitration was quantified by counting the Ntyr-positive neurons in the sections 0.6 to 3.1 mm rostral to the epicenter at 0.5 mm intervals in MnTBAP- and saline-treated groups, and counts were compared. The overall comparison between the two treatment groups demonstrated that treatment by MnTBAP at 4 mg/kg significantly decreased the number of Ntyr-positive neurons in the ventral gray matter of the cord (P<0.001). The number of Ntyr-positive neurons in the MnTBAP-treated group was significantly decreased at 1.1 (1.6 ± 0.55, P=0.03), 1.6 (2.9 ± 0.11, P<0.001), 2.1 (7.7 ± 0.13, P<0.001), 2.6 (9.4 ± 0.29, P<0.001) and 3.1 (13.3 ± 0.31, P<0.001) mm compared with vehicle-treated sections at 1.1 (2.3 ± 0.65), 1.6 (5.2 ± 0.25), 2.1 (12.0 ± 0.32), 2.6 (13.0 ± 0.27), and 3.1 (15.9 ± 0.30) mm rostral to the epicenter (Figure 5, lower panel).

The number of immuno-positive neurons increased with distance from the epicenter; this is not due to more neurons being oxidized or nitrated at the longer distance than at the shorter distance from the epicenter. Rather it is simply because most neurons were lost near the epicenter, and more neurons survived farther from the injury center, providing a larger number of neurons to be oxidized and labeled as HNE-positive or Ntyr-positive neurons.

### MnTBAP versus MP on protection against neurological dysfunction after SCI

**Figure 6:**
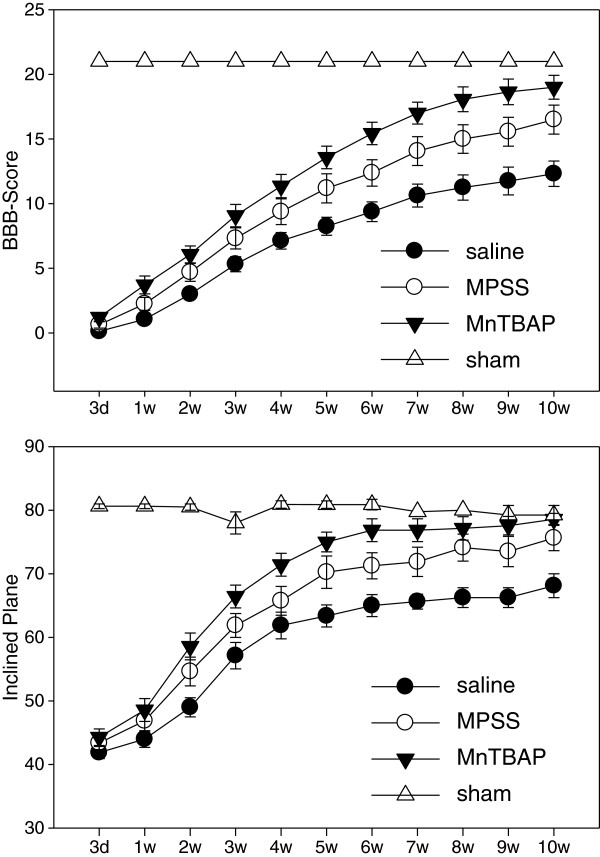
**MnTBAP versus MP on locomotor recovery following SCI as assessed by the BBB-test and inclined plane test. **The spinal cords of rats were injured at vertebra T10 (25 g.cm) using the NYU device. The behavioral tests were performed on animals treated with MnTBAP (ip), MPSS (iv), saline (ip), and a sham control group as described in the Methods section. The first dose of MnTBAP (10 mg/kg) or MPSS (30 mg/kg) were given at 4 h post-SCI and followed by one half of the first dose 2 h later. All experiments were triple blinded. **A**. results of BBB-test. **B**. results of inclined plane test. Error bars: mean ± SEM. Clearly, MnTBAP more effectively attenuates the neurological deficits on both tests than does MP.

SCI-induced neurological deficits were evaluated by the BBB test (Figure 6A) and the inclined plane test (Figure 6B) for 10 weeks. Behavioral test scores were compared by the two-way RMANOVA followed by all pairwise multiple comparisons between treatments: MnTBAP versus saline, MP versus saline, MnTBAP versus MP, and between treated and sham control. Post-SCI treatment with 10 mg/kg MnTBAP (ip) significantly increased BBB scores (p < 0.001) compared to saline-treated with no difference at 3 days (p=0.9) and significant differences at all other time points (p=0.03 for 1 week and p<0.001 for 2–10 weeks). MnTBAP also significantly increased the inclined plane angles (p<0.001) with no difference at 3 days and 1 week (p=1 and 0.8 respectively); significant differences started at 2 weeks (p=0.006 - 0.01 for 2–4 weeks and p<0.001 for 5–10 weeks). These results demonstrate that antioxidant therapy with MnTBAP significantly improved neurological dysfunction compared to the vehicle treatment and disproves the belief that antioxidant therapy is only effective shortly after SCI and of no value for longer term recovery. The standard regimen MPSS treatment also significantly improved neurological recovery (p<0.001 for both tests) compared to saline-treated. However there are no significant differences at any time point for both tests (p=0.2-1.0 for BBB and 0.5-1.0 for inclined plane tests). The scores in MnTBAP-treated animals are significantly better than MPSS-treated (p<0.001 for both tests). The significant differences started at 6 weeks (p=0.02 - 0.01) for the BBB test, with no difference at 3 days to 5 weeks (p=0.2-1.0). There were no difference at any time (p=0.2-1.0) for the inclined plane test. This demonstrates that 4 h post-SCI treatment with MnTBAP is more effective than 4 h post-SCI treatment with MPSS for improving neurological recovery after SCI. The scores for all treatment in injured animals were significantly worse compared to sham control (p<0.001 for all), indicating that pharmaceutical treatments alone cannot improve the dysfunction to a normal level.

## Discussion

In order to evaluate the potential of the catalytic antioxidant MnTBAP for the treatment of SCI, the present study characterized its abilities to scavenge ROS, to protect against oxidative stress in neurons and to ameliorate neurological dysfunction following SCI. The abilities to remove ROS and to improve functional recovery were compared between MnTBAP and MP, in addition to our previous comparison of the antioxidative and neuron protective effects between them [45] given that MP is the only drug used clinically for treating SCI.

By microdialysis or microcannula sampling and analysis of microdialysates or perfusates by HPLC or spectrophotometrically, we demonstrated that both H_2_O_2_ and O_2_^•-^ levels in the extracellular space of the rat spinal cord significantly increased following SCI (25 g.cm). The approximately 16% increase in average post-SCI H_2_O_2_ and the approximately 60% elevation of the average post-SCI O_2_^•─^ level compared with the pre-injury level are less than the 30% and 100% increases of H_2_O_2_ and O_2_^•-^ levels induced by a more severe injury (75 g.cm), as we reported previously [16,17]. Therefore, the production of H_2_O_2_ and that of O_2_^•-^ are positively significantly correlated with the severity of impact injury.

The abilities of MnTBAP and MP to reduce ROS were compared *in vivo* and *in vitro.* We demonstrated for the first time *in vivo* that the optimal dose of MnTBAP administered directly into the intrathecal space of the cord significantly reduced the elevation of H_2_O_2_ in the extracellular space to the basal level (Figure 1) and significantly reduced the elevation of O_2_^•-^ by approximately 50% following SCI, compared with the levels in the vehicle-treated animals (Figure 2). In contrast, the standard regimen of MPSS failed to significantly reduce the elevation of O_2_^•─^ (Figure 2). *In vitro* experiments were carried out to explore the possibly different mechanisms by which MnTBAP and MP attenuate concentrations of H_2_O_2_ and O_2_^•-^ (Figure 3). We demonstrated that treatment by MnTBAP significantly reduced H_2_O_2_ levels to approximately 40% of the levels in ACSF-treated samples, and significantly reduced O_2_^•-^ levels to approximately 80% of the levels in ACSF group. In contrast, MP had no effect on levels of either H_2_O_2_ or O_2_^•-^ compared with those of corresponding ACSF-treated groups. Our *in vitro* experiment demonstrated that MnTBAP can scavenge H_2_O_2_ and O_2_^•-^, whereas MP could not scavenge these ROS. Therefore the ROS-scavenging ability of MnTBAP should contribute to the reduction of SCI-elevated H_2_O_2_ and O_2_^•-^.

The catalase activity of most metalloporphyrins is less than 1% of that of native catalase [35]. The SOD activity of MnTBAP is 41 units/mg (the unit is defined as the amount of compound that inhibits the reduction of cytochrome c by 50%), and the catalase activity of MnTBAP is only 0.81/min (pseudo first-order rate constant for H_2_O_2_ decay) [58]. Our results demonstrated that with its “poor” catalase activity and greater SOD activity, MnTBAP was able to completely remove the overproduced H_2_O_2_ but only removed 50% of the overproduced O_2_^•-^*in vivo* in our SCI model. Given the optimal dose of MnTBAP (4 mg/kg), approximately 1 mg of MnTBAP was administered to a 250 g rat. Therefore, MnTBAP equivalent to 41 units of SOD activity was administered to a rat. According to this definition, if one unit inhibits the reduction of cytochrome c by 50%, then, 41 units should be much more than enough to inhibit all reduction of cytochrome c by O_2_^•-^. However, in our study 41 units inhibited the reduction of cytochrome c by only 50%, a forty fold lower SOD activity in our *in vivo* experiment than expected [58]. In contrast, the lower catalase activity of MnTBAP, as reported [58], brought the SCI-induced H_2_O_2_ elevation down to the basal level. Our *in vitro* experiment, consistent with our *in vivo* finding, also showed that MnTBAP proved more effective at scavenging H_2_O_2_ than it did O_2_^•-^ . Although the mechanism of the dramatic difference between the report [58] and both our *in vivo* and *in vitro* findings was not explored in this study, our results support the statement that “MnTBAP is not an SOD mimic, as it has negligible SOD-like activity” by Batinić-Haberle et al. [36].

The effectiveness of MnTBAP protection against oxidative stress was examined spatially around the injury epicenter. Because the neurons in the cord near the epicenter were killed immediately by acute mechanical injury, they could not be rescued by pharmaceutical intervention. Conversely, the cells far from the epicenter largely remained intact, so treatment should also not have much effect in this area, as reported [53]. Because substantial effects of the drug treatment can only be seen in a limited area, a spatial profile more accurately indicates the area of protection at the cellular level. Our spatial profiles indicated that 4 mg/kg of MnTBAP significantly reduced the numbers of HNE-positive neurons in the sections 1.55 to 2.55 mm rostral to the epicenter (Figure 4) and Ntyr-positive neurons in the sections 1.1 to 3.1 mm rostral to the epicenter (Figure 5) compared with numbers in the vehicle-treated group. MnTBAP at 4 mg/kg reduced MLP and protein nitration in a larger area than in our previous study [45] in which 1 mg/kg MnTBAP was also given intrathecally: 1 mm in length at 4 mg/kg and 0.5 mm long at 1 mg/kg for MLP, and 2.0 mm in length at 4 mg/kg and 0.5 mm at 1 mg/kg for protein nitration. The area of significant protection also started approximately 1 mm closer to the epicenter for 4 mg/kg than for 1 mg/kg. This comparison indicated that protective effects of MnTBAP are correlated with doses administered. The results in the present study together with our previous publications [44,45,53] indicated that MnTBAP ameliorates secondary damage within the boundary area. SCI treatment should be greatly improved by combining antioxidant therapy to save cells in the boundary area with other manipulations such as transplantation of stem cells, Schwann cells, and nanoparticles, etc. to restore the neurons and to repair and regeneration of axons in the injury epicenter. [59-61].

RS can cause injury by directly oxidizing the major cellular components. Sulfhydryl group in proteins is very susceptible to oxidation, nitration, nitrosation, and nitrosylation. Since the oxidative defense enzymes, such as SODs, catalase, GPx, thioredoxin, etc., are all sulfhydryl-containing enzymes, they may suffer function-distorting oxidative modification. MnTBAP scavenges SCI-induced RS, thereby protecting against oxidation and nitration of these antioxidant enzymes – helping restoration of their enzymatic activity. Functional recovery of defense enzymes, in turn, further reduces RS production. This positive feedback mechanism enhances the effects of MnTBAP protection. RS can cause injury by acting as intracellular death signals that lead to changes in expression of proteins, and by acting as modulators of the redox state. RS also have physiological functions. In redox cycles, the primary signal transducers are not large proteins but small redox-active molecules. ROS (e.g. O_2_^•─^, H_2_O_2_), and RNS (e.g. nitric oxide, S-nitrosothiols and ONOO^-^) can mediate redox signaling [62]. Therefore, in addition to catalytically scavenging a wide range of RS, other mechanisms such as modulating RS-based redox signaling pathways, and regulating cellular transcription activity may also contribute to the beneficial effects of metalloporphyrins [36]. It was reported that MnTBAP attenuated the nuclear translocation of apoptosis-inducing factor and the subsequent DNA fragmentation induced by ROS after permanent focal cerebral ischemia in mice [63]. So, in addition to their enzymatic catalyzing scavenging activities, more complicated mechanisms may be involved in our *in vivo* finding that MnTBAP reduced elevation of deleterious ROS, attenuated oxidative stress in neurons, and improved functional recovery after SCI.

The mechanisms by which the steroid MP protect against MLP and neurological deficits lies in its anti-oxidative and anti-inflammatory activities. In contrast to the effects of MnTBAP, our *in vitro* results demonstrated that MP had no effect on the levels of H_2_O_2_ and O_2_^•-^. This is consistent with the published report that MP does not directly trap radicals [64]. The activity of the oxidative defense enzyme GPx in erythrocytes of the rat was inhibited after SCI and MP restored GPx activity to near-basal levels [65]. Similar tendencies were also found for the activities of catalase and GPx in the spinal cord tissues of rats following SCI and MP treatment [66]. This suggests that the *in vivo* reduction of H_2_O_2_ by MP following SCI probably occurs indirectly through pathways, such as anti-inflammatory activities [67] and restoration of the activities of oxidative defense enzymes [65,66]. Our previous study demonstrated that MP significantly reduced the extracellular release of prostaglandin F_2α_ (PGF_2α_), a membrane hydrolysis product, following SCI. It blocked PGF_2α_-induced ^•^OH and MDA production. However, unlike MnTBAP, MP did not significantly reduce Fenton’s reagent-induced MDA production in the rat spinal cord, even though the MDA concentration was below the concentration induced by PGF_2α_. This suggests that 1) MP cannot directly scavenge ^•^OH, 2) its “antioxidant” effect is probably a consequence of interrupting the production of toxic hydrolysis products, and 3) the MLP is triggered by hydrolysis product-induced free radicals [68]. Therefore, the broad-spectrum RS scavenger MnTBAP better attenuates oxidative damage than does MP.

We previously measured the time course of protein nitration by counting the Ntyr-positive cells in sections stained by the anti-Ntyr antibody at different times post-SCI [20]. We found that protein nitration peaked at 12 to 24 h post-SCI. In a recently published study, Carrico et al. reported that Ntyr-positive and HNE-positive staining increased starting from 3 h to as long as 1 and 2 weeks post-SCI [26]. Christie et al. reported that after acute SCI the levels of MDA rose quickly at 4 h, returned to baseline at 12 h, and increased again by 24 h post-SCI, and the elevation was sustained for 120 h post-SCI [28]. Luo et al. reported that HNE-protein adducts increased in the damaged cord as early as 4 h after SCI, reached a peak level at 24 h, and remained significantly elevated up to 7 days after SCI [69]. Therefore, in the present study, the spatial profiles of MnTBAP protection against oxidative stress were measured at 24 h post-SCI. This time point was within the peak region of protein nitration, and production of MDA and HNE-protein adducts.

Although the regimen of MPSS applied in our study was the standard one for the clinical treatment of SCI, it was originally developed by using experimental animals. This regimen was reported as the optimal dose in cats compared with 15 and 60 mg/kg [70]. The bolus dose of 30 mg/kg plus follow-up administration was also used in experimental SCI of rats [71-73]. In a dose–response study using a ventral compression injury model, the best results were yielded when MPSS was administered in the rats with a bolus dose of 30 mg/kg at 30 min post-SCI followed by a second injection of 15 mg/kg (a total of 45 mg/kg) or with a 60 mg/kg bolus at 30 min post-SCI [74]. This demonstrates that the standard regimen of MPSS for the clinical treatment of SCI is also suitable for rats. Therefore, the standard clinical regimen of MPSS was applied in the present study.

Administering MP within 8 hours improves neurological recovery in SCI. However, the high dose of MP is ineffective and possibly even deleterious when given more than 8 hours post-SCI [43,75,76]. This indicates that 8 h is an effective time window for MP treatment. In many studies of experimental SCI treated with MP, MPSS was given immediately following SCI (0 h post-SCI). Unfortunately, in clinical practice patients can rarely be treated at 0 h post-injury. We demonstrated that the time window for MnTBAP to reduce oxidative damage and neuron death was at least 12 h after SCI [44]. Therefore, in addition to comparing the efficacy of 4 h post-SCI treatment with MnTBAP and MP to protect against oxidative stress and cell death as we reported [45], the present study further compared the effectiveness of 4 h post-SCI treatment with MnTBAP and MP in functional recovery after SCI. This is not only within the effective time window for both MP and MnTBAP, but also a more practical time window for clinical usage. We demonstrated that post-SCI treatment with 10 mg/kg MnTBAP (ip), or with the standard MP regimen (iv), both significantly increased BBB and inclined plane scores compared to vehicle treatment. However, the scores in MnTBAP-treated animals were significantly better than in MP-treated animals, demonstrating that post-SCI treatment with MnTBAP is more effective than MP for improving neurological dysfunction after SCI.

MnTBAP was reported as poorly penetrating the blood brain barrier [49], so it is not a favorable candidate for antioxidant therapy for central nervous system injury. We also demonstrated that the MP’s blood–spinal cord barrier penetrating ratio was much higher than MnTBAP (20% versus 4%) [50]. However, our results of behavioral tests demonstrated that with lower ability of penetrating the blood–spinal cord barrier, MnTBAP reached appropriate targets and more effectively improved the functional recovery than the standard treatment of SCI by MP, further supporting the candidacy of MnTBAP in treating SCI.

## Conclusion

1) By microdialysis or microcannula sampling and biochemical analysis, the present study demonstrated for the first time *in vivo* in a rat SCI model that the intrathecal optimal dose of MnTBAP significantly reduced the elevation of both H_2_O_2_ and O_2_^•-^ in the extracellular space of the rat spinal cord following SCI. In contrast, the standard regimen of MPSS failed to significantly reduce the elevation of O_2_^•─^ compared with levels in vehicle-treated animals. Our *in vitro* results demonstrated that MnTBAP can scavenge both H_2_O_2_ and O_2_^•-^, but showed that MPSS has no ability to remove either. 2) By measuring the spatial profile of MnTBAP protection against MLP and protein nitration in neurons, this study established the effective areas for the optimal dose of MnTBAP protection to reduce oxidative damage after SCI. 3) By behavioral tests, this study demonstrated for the first time that 4 h post-SCI treatment with MnTBAP (10 mg/kg, ip) was significantly more effective in reducing neurological dysfunction than the standard regimen of MP (iv). This is a most important criterion in determining the therapeutic potential of a candidate agent for SCI treatment. These results provide evidence that the generation of ROS during the secondary damage cascade after SCI is a cardinal factor leading to oxidative damage to neurons, thereby causing neurological dysfunction. ROS catalytic scavenging ability of MnTBAP is likely a major factor in its protection against oxidative damage and neurological dysfunction, possibly by a positive feedback mechanism: MnTBAP removes ROS, thereby reducing ROS-induced oxidative modification to the major cellular components, including the antioxidative defense enzymes. Activity restoration of these defense enzymes by MnTBAP, in turn, further reduces ROS production. This positive feedback mechanism would enhance the effects of MnTBAP protection. Combination of the findings in the present study and our previous publications [44,45] ranks the broad spectrum scavenger MnTBAP as superior to MP in removing ROS, thereby preventing oxidative damage to cellular components and so ameliorating neurological recovery. Our results suggest that MnTBAP is a potential candidate for antioxidant therapy following SCI.

## Abbreviations

ANOVA: Analysis of variance; ACSF: Artificial cerebrospinal fluid; BBB-test: Basso-Beattie-Bresnahan locomotor rating scale; 2,3-DHBA: 2,3-Dihydroxybenzoic acid; 2,5-DHBA: 2,5-Dihydroxybenzoic acid; GPx: Glutathione peroxidase; H2O2: Hydrogen peroxide; HNE: 4-hydroxy-nonenal; HPLC: High-pressure liquid chromatography; Ip: Intraperitoneally; Iv: Intravenously; MDA: Malondialdehyde; MnTBAP: Mn (III) tetrakis (4-benzoic acid) porphyrin; MLP: Membrane lipid peroxidation; MP: Methylprednisolone; MPSS: Methylprednisolone sodium succinate; NO: Nitric oxide; NSE: Neuron-specific enolase; Ntyr: Nitrotyrosine; NYU: New York University; O2•-: Superoxide anion; •OH: Hydroxyl radical; ONOO-: Peroxynitrite; PBS: Phosphate-buffered saline; PE: Polyethylene; PGF2α: Prostaglandin F_2α_; RMANOVA: Repeated measures of analysis of variance; RNS: Reactive nitrogen species; ROS: Reactive oxygen species; RS: Reactive species; s.c: Subcutaneously; SCI: Spinal cord injury; SOD: Superoxide dismutase.

## Competing interests

No competing financial and non-financial interests exist.

## Authors’ contributions

DL, the Principal Investigator, conceived the study, designed the experiments, trained the postdoctoral fellows and oversaw their work, analyzed and developed the presentation of the data, participated in the experiments of triple blinded behavioral tests, and wrote the entire manuscript. YS, a postdoctoral fellow, carried out all experiments for measuring the time courses of the production of ROS and the effect of MnTBAP on ROS production by microdialysis and microcannula sampling and by HPLC analysis, prepared figures, performed statistical analysis of data, and participated in the preparation of the manuscript for the portion of ROS production. LV, a postdoctoral fellow, carried out all experiments for characterizing oxidative stress in neurons and the effect of MnTBAP on oxidative stress levels, prepared figures, performed statistical analysis of data, and participated in the experiment of behavioral tests. FB, a postdoctoral fellow, carried out behavioral tests, prepared figures, performed statistical analysis of data for behavioral tests, and joined preparation of the manuscript for the portion of behavioral tests. All authors read and approved the final manuscript.
